# Myocardial extracellular volume is a non-invasive tissue marker of heart failure in patients with transposition of the great arteries and systemic right ventricle

**DOI:** 10.3389/fped.2022.949078

**Published:** 2022-11-07

**Authors:** Nadya Al-Wakeel-Marquard, Tiago Ferreira da Silva, Felix Berger, Titus Kuehne, Daniel R. Messroghli

**Affiliations:** ^1^ Department of Congenital Heart Disease – Pediatric Cardiology, German Heart Center Berlin, Berlin, Germany; ^2^ Institute of Computer-assisted Cardiovascular Medicine, Charité – Universitätsmedizin Berlin, corporate member of Freie Universität Berlin, Humboldt-Universität zu Berlin, and Berlin Institute of Health, Berlin, Germany; ^3^ DZHK (German Centre for Cardiovascular Research), partner site Berlin, Berlin, Germany; ^4^ Department of Pediatrics, Division of Cardiology, Charité – Universitätsmedizin Berlin, corporate member of Freie Universität Berlin, Humboldt-Universität zu Berlin, and Berlin Institute of Health, Berlin, Germany; ^5^ Department of Internal Medicine – Cardiology, German Heart Center Berlin, Berlin, Germany; ^6^ Department of Cardiology, Charité – Universitätsmedizin Berlin, corporate member of Freie Universität Berlin, Humboldt-Universität zu Berlin, and Berlin Institute of Health, Berlin, Germany

**Keywords:** transposition of the great arteries, systemic right ventricle, cardiovascular magnetic resonance, extracellular volume, myocardial fibrosis, heart failure

## Abstract

**Background:**

Focal myocardial fibrosis in the systemic right ventricle (RV) is related to ventricular dysfunction and adverse outcome in patients with d-transposition of the great arteries (dTGA) post atrial redirection and those with congenitally corrected TGA (ccTGA). The role of diffuse fibrotic lesions in these conditions remains poorly understood. Our study aimed to investigate diffuse myocardial fibrosis by measuring extracellular volume (ECV) with cardiovascular magnetic resonance (CMR) and to explore correlations between ECV and clinical as well as functional markers of heart failure in patients with TGA and systemic RV.

**Methods:**

We prospectively included dTGA and ccTGA patients aged ≥14 years and compared them to healthy controls. Standardized CMR included modified Look-Locker Inversion recovery T1 mapping to quantify diffuse myocardial fibrosis in the systemic RV and the subpulmonary left ventricle (LV). The centerline of RV and LV myocardium was marked with a line of interest tool to determine native and post-contrast T1 for quantification of ECV.

**Results:**

In total, 13 patients (dTGA: *n*  =  8, ccTGA: *n* = 5) with a median age of 30.3 years were enrolled. LV ECV was higher in patients than in controls [34% (30%–41%) vs. 26% (23%–27%), *p* < 0.001], with values increased above the upper limit of normal in 10/13 patients (77%). RV ECV tended to be higher in patients than in controls, albeit without statistical significance [29% (27%–32%) vs. 28% (26%–29%), *p* = 0.316]. Patients with elevated LV ECV had lower LV ejection fraction than those with normal ECV (52 ± 5% vs. 65 ± 4%, *p* = 0.007). Correlations with clinical parameters were not observed. LV ECV was significantly higher than RV ECV (*p* = 0.016) in the patient group.

**Conclusions:**

In this study, LV ECV was significantly increased in TGA patients compared to controls, and was associated with LV dysfunction. Our data suggest that ECV may serve as a non-invasive tissue marker of heart failure in TGA with systemic RV. Further research is necessary to evaluate the prognostic implications and the potential role of ECV in monitoring disease progression and guiding therapy, aiming to maintain LV function or train the LV for subaortic location in TGA patients from infancy to adulthood.

## Introduction

Given the advances in care for patients with congenital heart disease (CHD), the majority of children born with CHD reach adulthood (1, 2). However, increased mortality has been described in adult patients with CHD, particularly in the young, with heart failure as the leading cause of death (3). Heart failure signs and symptoms are seen in 22% of patients with d-loop transposition of the great arteries (dTGA) post atrial redirection, and in 32% of patients with congenitally corrected transposition of the great arteries (ccTGA) (4).

Myocardial fibrosis contributes to the development of heart failure in CHD (5, 6). Cardiovascular magnetic resonance (CMR) studies have identified focal myocardial fibrosis in the systemic right ventricle (RV) as assessed by late gadolinium enhancement (LGE) imaging to be associated with ventricular dysfunction and adverse outcome in dTGA post atrial redirection and in ccTGA (7–10). Data on diffuse myocardial fibrosis from CMR T1 mapping in TGA with a systemic RV are limited and somewhat controversial, pointing to an increased fibrotic burden in the systemic RV (11), the subpulmonary left ventricle (LV) (12), the interventricular septum (13), or both RV and LV (14).

With the present study, we aimed to determine biventricular extracellular volume (ECV) as a non-invasive measure of diffuse myocardial fibrosis by applying CMR T1 mapping and to evaluate the correlation of ECV with clinical and functional markers of heart failure in patients with TGA and systemic RV.

## Materials and methods

### Study population

We prospectively enrolled patients of ages ≥14 years with a diagnosis of dTGA post atrial redirection or ccTGA who were referred for clinically indicated CMR. Exclusion criteria were arrhythmias, cardiac decompensation, cardiomyopathies, chronic or acute infection up to four weeks prior to CMR, chronic systemic diseases, significant renal impairment, devices non-compatible with CMR, and claustrophobia. Patients further underwent blood sampling for hematocrit and N-terminal pro brain natriuretic peptide (NT-proBNP) immediately before CMR, and cardiopulmonary exercise testing (CPET).

Healthy subjects with normal 12-lead electrocardiogram (ECG), blood pressure, and renal function, and no history or symptoms of cardiovascular disease or contraindications for CMR were included as controls.

### CMR protocol

All participants received standardized CMR at 1.5 T (Philips Healthcare, Best, The Netherlands). Blood samples for hematocrit and NT-proBNP were collected immediately before CMR. Cine images were acquired with a steady-state free precision sequence in short axis (SAX) and long axis planes as well as in axial orientation covering the entire ventricles. LGE imaging in SAX was conducted using a T1-weighted inversion-recovery three-dimensional spoiled gradient echo sequence 5 min after intravenous bolus application of gadolinium-DOTA (Dotarem®, Guerbet) at a dose of 0.1 mmol/kg. An individually adapted prepulse delay Look-Locker sequence was applied to determine inversion times for nulling myocardium.

**Figure 1:**
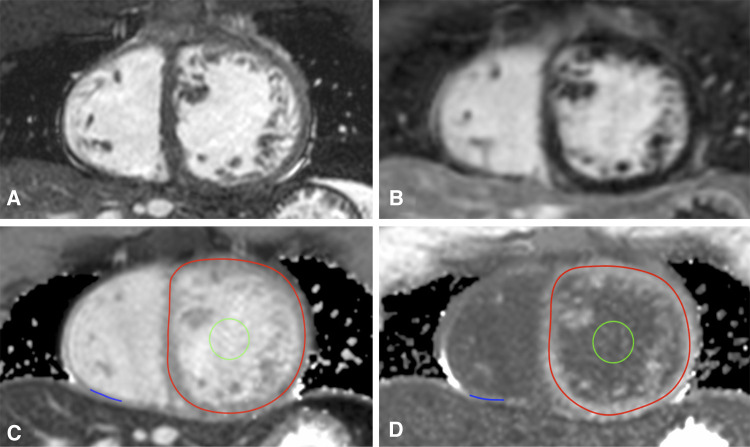
T1 mapping in congenitally corrected transposition of the great arteries. Corresponding cine (**A**) and late gadolinium enhancement images (**B**) as well as T1 maps in midventricular short axis before (**C**) and after contrast application (**D**) are shown. Lines of interest (LOIs) are drawn into the myocardium of the systemic right ventricle (red LOI) and the subpulmonary left ventricle (blue LOI). The blood pool is marked with a green region of interest.

For T1 mapping, an ECG-gated single-shot modified Look-Locker inversion-recovery (MOLLI) sequence was used (15) with sequence parameters as follows: MOLLI scheme 3b(3b)3b(3b)5b, slice thickness 8.0 mm, repetition time 2.4 ms, echo time 1.2 ms, flip angle 35°. Native and post-contrast T1 images were acquired in midventricular SAX and axial four-chamber orientation in breath-hold at end-expiration before and 15 min after contrast bolus application (Figure 1).

### CMR analysis

A commercially available workstation (Philips Viewforum, Best, The Netherlands) was used to analyze LV and RV volumes [end-diastolic volume (EDV), end-systolic volume (ESV), stroke volume (SV)] and ejection fraction (EF) from cine image stacks in axial orientation. Volumetric parameters were indexed to body surface area (BSA). In TGA patients, the ventricle in the subpulmonary position was defined as the LV, and the ventricle in the subaortic position as the (systemic) RV.

T1 maps were generated with the open-source image reconstruction tool MRmap after manual correction for body motion (16), stored as DICOM files, and analyzed with OsiriX Lite. Native and post-contrast blood T1 values were gained from a region of interest placed in the blood pool of the systemic ventricle in subaortic location with exclusion of the papillary muscles and the trabeculae, respectively. For measurements of myocardial T1, a line of interest (LOI) was manually drawn in the center of the RV and LV myocardium as previously described (17). LOIs marked the myocardial circumference of the subaortic ventricle and the thickest part of the free, anterior or inferior wall of the subpulmonary ventricle on T1 maps in midventricular SAX (Figure 1). In the case of better delineation of myocardial walls, T1 maps in four-chamber-view were alternatively used. Native and post-contrast T1 values of myocardium and blood as well as hematocrit were then used to calculate RV ECV and LV ECV. ECV was considered to be elevated at values above the mean plus two standard deviations (SD) of the control ECV (18).

### Follow-up

Medical records of TGA patients were reviewed for adverse events since the date of CMR, including supraventricular and ventricular arrhythmias, implantation of a pacemaker or implantable cardioverter defibrillator (ICD), heart failure-related hospitalization, heart transplantation (HTx), and cardiovascular death.

### Statistical analysis

Statistical analyses were carried out with SPSS version 25.0 (IBM Corp., Armonk, NY, United States), considering *p*-values <0.05 to be statistically significant. Data between groups were compared with the Mann–Whitney *U* test, and within groups using Wilcoxon signed rank test with Bonferroni correction, respectively. For comparisons between categorical variables, Fisher’s exact test was applied. Spearman's correlation coefficient was used to analyze associations between continuous variables. Results are presented as median (range), mean ± SD, or numbers (*n*) and percentages (%), as appropriate.

## Results

### Subject characteristics

In total, 13 patients with a systemic RV and a median age of 30.3 (25.2–37.9) years were enrolled. Of those, eight patients had dTGA post atrial redirection (Senning procedure: *n* = 7, Mustard procedure: *n* = 1), and five had ccTGA. A summary of patient characteristics is given in Table 1. Healthy controls (*n* = 15; female: *n* = 7) had a median age of 24.4 (23.8–29.0) years and were younger than the patient group (*p* = 0.004). Differences in gender distribution were not seen.

**Table 1:** Patient characteristics.

	Patients total (*n* = 13)	dTGA Senning/Mustard (*n* = 8)	ccTGA (*n* = 5)	*p*-value
Gender (female/male)	9 (69)/4 (31)	5 (62.5)/3 (37.5)	4 (80)/1 (20)	1.000
Age at CMR (years)	30.3 (25.2–37.9)	32.2 (29.1–37.3)	25.0 (23.0–41.5)	0.524
BSA (kg/m²)	1.9 (1.9–2.0)	2.0 (1.7–2.2)	1.9 (1.9–2.0)	0.524
NYHA				
I	7 (54)	3 (37.5)	4 (80)	0.266
II	6 (46)	5 (62.5)	1 (20)	
III	0 (0)	0 (0)	0 (0)	
IV	0 (0)	0 (0)	0	
VO_2_max (ml/min/kg)	22.3 (15.8–30.0)	20.4 (13.9–22.8)	25.6^a^	0.071
	*n* = 8	*n* = 6	*n* = 2	
NT-proBNP (pg/ml)	236.3 (179.9–662.8)	247.4 (183.1–850.2)	198.8 (105.8–662.8)	0.622
Arrhythmias				
SVT	1 (8)	1 (14)	0 (0)	1.000
nsVT	2 (17)	2 (29)	0 (0)	0.470
	*n* = 12	*n* = 7		
Pacemaker	1 (8)	0 (0)	1 (80)	0.417
ICD	2 (17)	2 (29)	0 (0)	0.470
	*n* = 12	*n* = 7		
MCS	0 (0)	0 (0)	0 (0)	n.a.
HTx	1 (8)	0 (0)	1 (20)	0.417
Death	1 (8)	1 (14)	0 (0)	1.000
	*n* = 12	*n* = 7		
CMR findings				
LVEF (%)	55 (49–62)	50 (47–62)	56 (52–62)	0.435
LVEDV (mL/m²)	68 (52–90)	59 (47–67)	94 (77–105)	**0.006**
LVESV (mL/m²)	35 (24–39)	28 (21–35)	41 (32–45)	**0.045**
LVSV (mL/m²)	32 (30–51)	31 (27–32)	51 (42–63)	**0.011**
RVEF (%)	45 (34–55)	43 (31–56)	46 (39–54)	0.622
RVEDV (mL/m²)	113 (97–159)	106 (92–155)	128 (109–203)	0.284
RVESV (mL/m²)	67 (45–96)	63 (43–104)	70 (53–123)	0.435
RVSV (mL/m²)	53 (45–60)	47 (37–54)	60 (55–79)	**0.030**
Presence of LGE	9 (75)^b^	7 (100)	2 (40)	**0.045**

Values are median (range) or n (%). Statistically significant p-values are indicated in bold.

BSA, body surface area; ccTGA, congenitally corrected transposition of the great arteries; CMR, cardiovascular magnetic resonance; dTGA, d-loop transposition of the great arteries; HTx, heart transplantation; ICD, implantable cardioverter defibrillator; LGE, late gadolinium enhancement; LVEDV, left ventricular end-diastolic volume; LVEF, left ventricular ejection fraction; LVESV, left ventricular end-systolic volume; LVSV, left ventricular stroke volume; MCS, mechanical circulatory support; nsVT, non-sustained ventricular tachycardia; NT-proBNP, N-terminal pro brain natriuretic peptide; NYHA, New York Heart Association; RVEDV, right ventricular end-diastolic volume; RVEF, right ventricular ejection fraction; RVESV, right ventricular end-systolic volume; RVSV, right ventricular stroke volume; SVT, supraventricular tachycardia; VO_2_max, maximum oxygen consumption.

^a^ Median value was given.

^b^ LGE image quality was insufficient in one patient.

### Cardiovascular magnetic resonance

#### Late gadolinium enhancement

Mild LGE was visible in 75% of the patients (dTGA post Senning: *n* = 7, ccTGA: *n* = 2), and was located at the insertion points (*n* = 5), the inferior (*n* = 2) or anterior RV wall (*n* = 1), and the septum (*n* = 1). Transmural LGE was not observed.

#### Extracellular volume

LV ECV was higher in patients than in controls [34% (30%–41%) vs. 26% (23%–27%), *p* < 0.001; Table 2]. Values were increased above the upper limit of normal (>31.5%) in 10/13 patients (77%). In two out of three dTGA patients with normal LV ECV, (sub-) pulmonary stenosis was present, and the remaining patient had ccTGA without stenosis. RV ECV tended to be higher in patients than in controls, yet without statistical significance [29% (27%–32%) vs. 28% (26%–29%), *p* = 0.316]. Elevated RV ECV (>29.8%) was detected in four patients (31%). LV ECV was significantly higher than RV ECV (*p* = 0.016) in the patient group. There were no differences in LV ECV and RV ECV between dTGA and ccTGA patients.

**Table 2:** T1 mapping parameters in controls and patients.

	Controls (*n* = 15)	Patients (*n* = 13)	*p*-value
LV T1 native (ms)	989 (966–998)	1023 (978–1059)	0.072
LV ECV (%)	26 (23–27)	34 (30–41)	<**0.001**
RV T1 native (ms)	979 (953–1027)	1002 (978–1054)	0.235
RV ECV (%)	28 (26–29)	29 (27–32)	0.316

Values are median (range). Statistically significant p-values are indicated in bold.

LV T1 native, left ventricular native T1; LV ECV, left ventricular extracellular volume; RV T1 native, right ventricular native T1; RV ECV, right ventricular extracellular volume.

##### ECV and clinical data

LV ECV and RV ECV of patients did not correlate with clinical characteristics including age at CMR, BSA, systolic or diastolic blood pressure, NT-proBNP, maximum oxygen consumption from CPET, and gender distribution.

##### ECV and CMR measures

**Figure 2:**
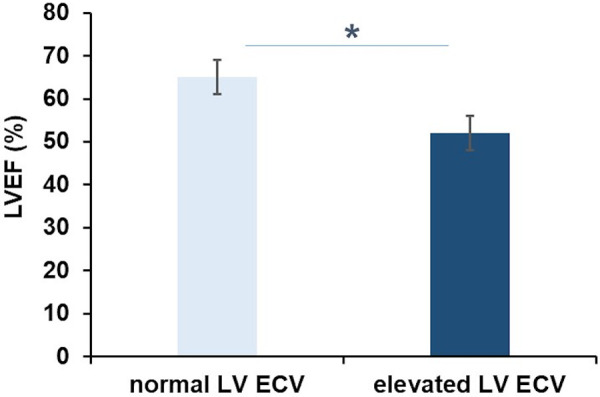
Extracellular volume and left ventricular function in transposition of the great arteries. Patients with elevated left ventricular extracellular volume (LV ECV) had significantly lower LV ejection fraction (LVEF) than patients with normal LV ECV (52 ± 5 vs. 65 ± 4%; *p = 0.007).

Patients with elevated LV ECV had significantly lower LVEF than those with values within the normal range (52 ± 5% vs. 65 ± 4%, *p* = 0.007; Figure 2). Both LVEF and RVEF were reduced in patients with elevated RV ECV as compared to those with normal values, but these differences were not statistically significant (51 ± 2% vs. 56 ± 3%, and 39 ± 5% vs. 46 ± 4%, *p* = 0.371, respectively). LV ECV and RV ECV did not differ between patients with and those without LGE (*p* = 0.482 and 0.145, respectively). Among patients with positive LGE in the systemic RV, 67% (*n* = 6) had elevated LV ECV (*p* = 0.509). There were no differences in the presence of LGE between those with elevated RV ECV and those without (*p* = 0.509).

### Follow-up

Median follow-up time was 5.1 (2.5–8.2) years. One patient was lost to follow-up. Adverse events were observed in 6/12 patients (50%). Of those, four patients (67%) had elevated LV ECV, with additionally elevated RV ECV in two patients. Among those with dTGA post Senning and elevated LV ECV, one patient had isolated non-sustained ventricular tachycardia documented during Holter-ECG. The two patients with elevated LV ECV and RV ECV developed severe dysfunction of the systemic RV and underwent ICD implantation, in one followed by recurrent atrial and ventricular arrhythmias and progressing heart failure, hospitalization, and cardiovascular death 4.0 years after CMR. One patient with ccTGA and elevated LV ECV developed severe heart failure leading to hospitalization and HTx. In those with normal LV ECV, adverse events included hospitalization due to cardiac decompensation in one patient with dTGA post Senning, and complete heart block requiring pacemaker implantation in one patient with ccTGA.

## Discussion

In our study, increased LV ECV in TGA patients as compared to controls indicates the presence of diffuse myocardial fibrosis in the subpulmonary LV in the setting of a systemic RV. LV ECV was elevated above the upper limit of normal in the majority of TGA patients and was associated with a reduction in LV function. LV ECV was higher than RV ECV in this cohort.

It has been shown that focal myocardial fibrosis visualized with LGE imaging in the systemic RV correlates with ventricular dysfunction, cardiac arrhythmias, exercise intolerance, and clinical deterioration in patients with dTGA post atrial redirection (7–10) and ccTGA (8). However, the ability of LGE to assess diffuse fibrotic lesions is restricted, and only a few studies have reported on CMR T1 mapping-derived measures of diffuse myocardial fibrosis in TGA (11–14). In the study by Plymen et al., septal ECV was significantly higher in 14 dTGA patients post Senning or Mustard procedure than in age- and gender-matched controls, and was positively related to NT-proBNP and negatively to the chronotropic index during CPET. ECV of the RV wall was not measured for technical reasons (13). Broberg et al. found significantly higher levels of diffuse myocardial fibrosis in association with NT-proBNP in the systemic RV of 53 patients with dTGA post atrial redirection or ccTGA. In that cohort, elevated RV ECV was associated with adverse clinical outcomes, suggesting a role of fibrosis in the development of heart failure (11). Both studies did not report ECV of the subpulmonary LV (11, 13). We evaluated diffuse myocardial fibrosis in the systemic RV as well as in the subpulmonary LV and found significantly higher LV ECV in TGA patients as compared to controls. The majority of patients (77%) had elevated LV ECV and had significantly lower LVEF than those with normal ECV values. Given that conventional heart failure therapies generally fail to improve RV function significantly (19), we anticipate that our findings will stimulate further research on the impact of the LV on morbidity and mortality, and on its role as a potential therapeutic target. The assessment of diffuse myocardial fibrosis by ECV may be useful in selecting patients who may potentially benefit from pulmonary artery banding by improving RV function and tricuspid regurgitation, thus bridging to transplantation or delaying listing for transplantation (20), or by training the LV of ccTGA patients for double switch operation. Furthermore, in our patient group, LV ECV was significantly higher than RV ECV. This is in line with Shehu et al. and Cheung et al. describing greater LV ECV than RV ECV in 10 and 31 dTGA patients post Senning or Mustard, respectively (12, 14). We agree with Cheung et al. that the finding of a higher fibrotic burden in the subpulmonary LV than in the systemic RV is intriguing and that the cause is not immediately apparent. Chronic volume unloading of the eccentrically compressed LV in relation to septal shift and LV diastolic dysfunction, and postcapillary pulmonary hypertension have been discussed as potential contributors, yet the role of these factors in the development of LV fibrotic remodeling remains to be studied further (14).

Cheung et al. in addition to elevated LV ECV also detected higher RV ECV values in the TGA cohort, although with greater involvement of the subpulmonary LV (14). In our study, a trend toward higher RV ECV in patients than in controls was seen, albeit without statistical significance. We found RV ECV to be elevated in almost one-third of the patients (31%). Both RVEF and LVEF were lower than in those with normal RV ECV values, but again these differences were not statistically significant. Apart from technical-methodological differences, the younger age of our TGA cohort in comparison to the abovementioned work by Broberg et al. and Cheung et al. may be contributive to the discrepancies in RV ECV between the studies. Furthermore, we speculate that due to the relatively small sample size, our study may have been underpowered to achieve statistical significance with regard to the differences in RV ECV between patients and controls and the association with impaired biventricular function. Future long-term studies on larger cohorts are required to substantiate our speculations and explore the role of age in the development of diffuse myocardial fibrosis in TGA.

Interestingly, we saw both LV ECV and RV ECV to be elevated in the two dTGA patients requiring ICD implantation due to a severe decline in RV function during follow-up, followed by cardiovascular death in one patient. This points to the importance of interventricular interactions also on the extracellular matrix level, and in turn underlines the potential value of both systemic and subpulmonary myocardial ECV as non-invasive markers for prognosis, risk stratification, and monitoring of disease and therapy in TGA. Moreover, we suspect that the mild, but frequently present LGE in 75% of the TGA patients reflects fibrosis burden that may contribute to the RV dysfunction seen in our cohort. Among patients with positive LGE in the systemic RV, 67% had elevated LV ECV, again pointing to interactions between systemic and subpulmonary ventricles on the tissue level. However, our data in relation to LGE were not statistically significant, which may be explained by the limited number of patients included and the consequent lack of power. Therefore, further research to support our results and their prognostic implications is warranted. The importance of ventriculo-ventricular-interactions and the role of the subpulmonary LV not only as a contributor to morbidity and mortality but also as a potential therapeutic target in right-sided CHD has been well recognized (21). Further investigations need to address the use of ECV measurements for the management of disease and treatment with the goal of preserving LV function or training the LV for systemic pressure in subaortic position.

### Limitations

Our study is limited by the small sample size. Due to the lack of clinical indication for regular CMR examinations with contrast enhancement, longitudinal ECV data were unfortunately not available. Future long-term studies on larger cohorts to confirm our findings are required, and may potentially elucidate further differences between groups and associations between ECV and markers of heart failure. Healthy controls were younger compared to the patient group. However, an age-related increase in ECV is seen in individuals significantly older than the patients included in our study (22) and, thus, should not affect comparability between the two groups. Despite the application of the previously described LOI tool, especially helpful for T1 measurements in thin structures (17), and particular care at placing the LOI in the center of the myocardium of the respective subpulmonary ventricle, partial voluming with blood or fat may have caused potential overestimation of ECV. Given the lack of clinical indication for biopsy, we did not perform histological validation of our results.

## Conclusions

In this study, LV ECV was significantly higher in TGA patients than in controls and was associated with LV dysfunction. Our data suggest that ECV measurements may serve as an imaging marker of heart failure in the setting of TGA with a systemic RV. Further research is necessary to evaluate the prognostic implications and the potential role of ECV in non-invasively monitoring disease progression and guiding therapy, aiming to maintain LV function or train the LV for subaortic location in TGA patients from infancy to adulthood.

## Data Availability

The raw data supporting the conclusions of this article will be made available by the authors, without undue reservation.
